# Inverse Association between Canned Fish Consumption and Colorectal Cancer Risk: Analysis of Two Large Case–Control Studies

**DOI:** 10.3390/nu14081663

**Published:** 2022-04-16

**Authors:** Carlotta Franchi, Ilaria Ardoino, Cristina Bosetti, Eva Negri, Diego Serraino, Anna Crispo, Attilio Giacosa, Elena Fattore, Alberto Dolci, Francesca Bravi, Federica Turati, Carlo La Vecchia, Barbara D’Avanzo

**Affiliations:** 1 Department of Health Policy, Istituto di Ricerche Farmacologiche Mario Negri IRCCS, 20156 Milan, Italy; ilaria.ardoino@marionegri.it (I.A.); barbara.davanzo@marionegri.it (B.D.); 2 Italian Institute for Planetary Health (IIPH), 20156 Milan, Italy; 3 Department of Oncology, Istituto di Ricerche Farmacologiche Mario Negri IRCCS, 20156 Milan, Italy; cristina.bosetti@marionegri.it; 4 Department of Medical and Surgical Sciences, Alma Mater Studiorum, Università di Bologna, 40126 Bologna, Italy; eva.negri@unibo.it; 5 Unit of Cancer Epidemiology, Centro di Riferimento Oncologico, National Cancer Institute IRCCS, 33108 Aviano, Italy; serrainod@cro.it; 6 Epidemiology and Biostatistics Unit, Istituto Nazionale dei Tumori IRCCS, Fondazione G. Pascale, 80131 Naples, Italy; anna.crispo@tin.it; 7 Department of Gastroenterology and Clinical Nutrition, Policlinico di Monza, 20900 Monza, Italy; attilio.giacosa@gmail.com; 8 Department of Environmental Health Sciences, Istituto di Ricerche Farmacologiche Mario Negri IRCCS, 20156 Milan, Italy; elena.fattore@marionegri.it; 9 Research and Development Department, Bolton Food SpA, 20124 Milan, Italy; adolci@boltonfood.com; 10 Department of Clinical Sciences and Community Health, Università degli Studi di Milano, 20133 Milan, Italy; francesca.bravi@unimi.it (F.B.); federica.turati@unimi.it (F.T.); carlo.lavecchia@unimi.it (C.L.V.)

**Keywords:** primary prevention, nutrition, canned fish, colorectal cancer

## Abstract

Fish is among the foods exerting favourable effects on colorectal cancer (CRC), but the possible role of canned fish has been insufficiently investigated. We aimed to investigate the relationship between canned fish consumption and CRC risk. We analysed data from two case–control studies conducted between 1992 and 2010 in several Italian areas, comprising a total of 2419 incident cases and 4723 hospital controls. Canned fish consumption was analysed according to the weekly frequency of consumption as <1 serving per week (s/w) (reference category), 1 < 2 s/w, and ≥2 s/w. We calculated odds ratios (ORs) and 95% confidence intervals (CIs) using unconditional logistic regression models, adjusting for several recognised confounding factors. Overall, canned fish consumption was lower among cases than among controls (23.8% vs. 28.6%). An inverse association was found between canned fish consumption and CRC risk with a significant trend in risk (OR = 0.81, 95% CI: 0.71–0.92 for intermediate consumption and OR = 0.66, 95% CI: 0.51–0.85 for the highest one), which was consistent across strata of several covariates. This study is the first to offer a basis of support for canned fish consumption as a component of a healthy diet, and it has relevant public health implications given the high ranking of CRC in incidence and mortality worldwide.

## 1. Introduction

In 2020, colorectal cancer (CRC) accounted for 1.9 million new cases of cancer worldwide, ranking second for women and third for men in terms of incidence, and it was the second leading cause of cancer mortality, with 935,000 deaths [1]. High-income countries have the highest risk of CRC, but in countries undergoing major transition (i.e., Eastern Europe, South-Eastern and South-Central Asia, and South America), incidence rates tend to rise, likely reflecting changes in lifestyle factors and diet [1]. Modifiable risk factors for CRC are a sedentary lifestyle, leading to decreased physical activity and increased body weight; heavy alcohol consumption; tobacco smoking; and elevated consumption of red or processed meat [2]. The intake of whole grains, dietary fibre, dairy products and calcium, non-starchy vegetables and fruit has been inversely associated with CRC risk [2]. Fish has been shown to play a favourable role in cancer risk, especially in that of the digestive tract, including CRC, although the evidence remains inconsistent [3,4,5]. Fish is the main dietary source of long-chain omega-3 polyunsaturated fatty acids (PUFAs), which have anti-inflammatory properties and, consequently, exert anticarcinogenic effects [6,7]. Ecological studies showed mixed evidence of an association between omega-3 polyunsaturated fat intake and the risk of several cancers, sparking some debate on the topic [8,9].

In some studies, fish consumption comprises canned fish consumption, i.e., fish that has been steamed, cooled, cleaned, sealed in a can with covering of olive oil or brine, and then heated for a determined time for sterilisation in order to last up to several years [10]. Canned fish is rich in proteins and many other essential nutrients, such as omega-3 fatty acids [10].

Household consumption of processed fish and seafood, such as canned fish, has been increasing in recent decades, totalling 727,000 tons in 2018 [11]. In particular, a further increase in the consumption of canned fish in Europe was observed during the COVID-19 pandemic, likely because more people turned to home cooking and retail purchases and considered canned fish a practical, easy to preserve, ready to eat, affordable, and valuable substitute for fresh fish [11]. Nonetheless, to the best of our knowledge, the effect of canned fish consumption has not been adequately investigated in relation to cancer risk as a standalone item, separately from fresh fish.

This study aims to investigate the relationship between canned fish consumption and CRC risk, using data from two large case–control studies conducted in Italy.

## 2. Material and Methods

### 2.1. Study Population

Data for this study were obtained from two Italian case–control studies on CRC: the first study [12] was carried out in six Italian areas spanning North to South (i.e., Milan, Genoa, Pordenone, Gorizia, Forlì, Latina, and Naples) in the period of 1992–1996, and the second [13] was conducted in Milan, Pordenone, and Udine in 2008–2010. In both studies, cases were subjects with incident histologically confirmed CRC (ICD-9 codes: 153.*, 154.0-1), diagnosed no longer than 1 year prior to the interview and with no previous diagnoses of cancer at other sites. Controls were individuals with no history of cancer, who had not recently changed their diet, and who were admitted to the same hospitals as cases of acute, non-neoplastic conditions unrelated to hormonal or digestive tract diseases. There were 2723 and 4901 eligible cases and controls, respectively. Among those, a total of 2419 incident, histologically confirmed CRC cases and 4723 controls were included in this study: 1953 cases (57.6% males, median age 62, interquartile range (IQR): 55–68 years) and 4154 controls (49.9% males, median age 58, IQR: 48–65 years) from the first study, and 466 cases (65.9% males, median age 67, IQR: 60–72 years) and 569 controls (64.8% males, median age 66, IQR: 59–71 years) from the second study. Overall, there were 727 patients with cancer of the distal colon (splenic flexure, descending colon, sigmoid colon), 373 patients with cancer of the proximal colon (appendix, caecum, ascending colon, hepatic flexure, transverse colon), 426 with overlapping or not otherwise specified cancer of the colon, and 885 with cancer of the rectum (rectum and rectosigmoid junction); 8 patients had missing information on specific site.

Among controls, 1260 (26.7%) were admitted for non-traumatic orthopaedic disorders, 1134 (24.0%) for surgical conditions, 953 (20.2%) for trauma, 842 (17.8%) for eye diseases, and 534 (11.3%) for a miscellanea of other diseases. The study protocols were approved by the local ethical committees of the respective centres, and all participants signed an informed consent according to the rules at the time when the study was conducted.

### 2.2. Data Collection

Trained staff collected data through face-to-face interviews. The data include sociodemographic and anthropometric factors, physical activity, smoking habits, selected medical conditions, family history of CRC, and dietary habits in the 2 years before the interview. Subjects were also asked to report height and average weight at ages 30 and 50 years, as well as before diagnosis/interview. Occupational physical activity at ages 15–19, 30–39, and 50–59 years was self-reported as very heavy, heavy, intermediate, standing, or sedentary.

Usual diet was assessed through a structured, validated [14], and reproducible [15] food frequency questionnaire (FFQ), including information on weekly consumption of 78 foods in the first study and 56 foods in the second one, collected in six sub-sections. In particular, three items concerned the consumption of fish: one referred to boiled/grilled/baked fresh fish and shellfish, one referred to fried fish, and an additional one referred specifically to canned fish in olive oil (tuna, mackerel, sardines, etc.). A separate section investigated the history of consumption of alcoholic beverages. Occasional intake (no more than 3 times per month) was coded as 0.5 servings per week. An Italian food composition database [16], integrated with other sources, was used to estimate intake of total energy. The FFQ was tested for reproducibility [15] and validity using a 7-day dietary record as a reference method [14], giving a correlation coefficient of reproducibility of canned fish of 0.5 and a coefficient of validity of 0.5 for polyunsaturated fatty acids.

### 2.3. Statistical Analysis

Canned fish was analysed according to the weekly frequency of consumption as <1 serving per week (s/w) (used as reference category), 1 < 2 s/w, ≥2 s/w, and as a continuous variable where the average serving of canned fish in the Italian diet was 80 gr/day. Odds ratios (OR) and corresponding 95% confidence intervals (CI) were estimated using an unconditional multivariable logistic regression model [17]. Three hierarchical models were fitted for this purpose. The first regression model was adjusted by sex, age, centre, and study. In the second model, body mass index (BMI) before diagnosis (<25, 25–30, ≥30 Kg/m^2^), years of education (<7, 7–11, ≥12), and family history of cancer (yes/no) among first-degree relatives (parents, children, and siblings) were added. In the third model, tobacco smoking (never a smoker, former smoker since ≥1 year, current smoker of <15 cigarettes/day, and current smokers of ≥ 15 cigarettes/day), alcohol consumption (<1, 1–10, 10–21.5, >21.5 drinks/week), level of occupational physical activity at age 30–39 years (low, moderate, heavy), fruit (≥16.5, <16.5 s/w) and vegetable (≥11.5, <11.5 s/w) consumption, and total energy intake (≥2272, <2272 kcal/day) were also considered. The cut-off values for categories were identified based on the distribution of the variables among the controls. In the fourth model, fresh fish was added as a confounding factor. ORs and 95% CIs were also computed for type of fish consumption (no fish at all, only fresh, only canned, both types of fish) in a separate model.

Missing values for confounding variables were imputed with the most frequent categories, according to case/control and gender. A complete case analysis was also conducted. Tests for linear trend were performed by including exposure covariate as continuous in the model.

Stratified analyses were carried out according to sex; age; education; smoking; alcohol consumption; physical activity at the workplace; energy intake; and fruit, vegetable, and fresh fish consumption. Heterogeneity across strata was assessed for canned fish intake as a continuous variable (10 gr increase intake) using the Wald χ^2^ test with 1 degree of freedom.

All the analyses were carried out using SAS software 9.4 (SAS Institute Inc., Cary, NC, USA).

## 3. Results

Selected characteristics of 2419 CRC cases and 4723 controls are reported in Table 1. Cases were more frequently males, were slightly older, and reported a family history of intestinal cancer more frequently than controls.

**Figure 1:**
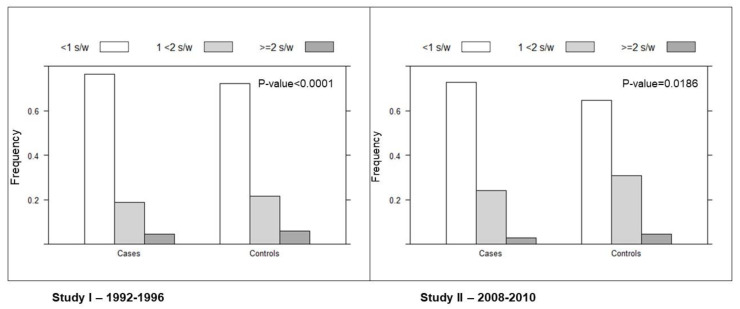
Distribution of canned fish consumption in cases and controls in the two studies.

Canned fish consumption in the cases and controls in the two studies are reported in Figure 1. Canned fish consumption was lower among cases than among controls (76.2% vs. 71.4%, respectively, did not consume canned fish or consumed it only occasionally). In the second study, compared to the first one, more patients consumed canned fish both among cases and controls: 23% of cases and 27.6% of controls consumed ≥1 s/w in the first study vs. 27.2% of cases and 35.3% of controls in the second study. The mean serving of canned fish was 5.61 gr/day among cases and 6.29 gr/day among controls, and this was similar in the two studies. Finally, in both studies, mean canned fish consumption was slightly lower in females (0.49 s/w) than in males (0.57 s/w) and in patients aged 60 or more (0.51 s/w) than in younger ones (0.56 s/w) in either cases or controls (data not shown).

Table 2 shows ORs for canned fish consumption. An inverse association was found between canned fish and CRC risk with a significant trend in risk. In particular, the ORs from model 4 were 0.81 (95% CI: 0.71–0.92) for intermediate consumption compared to the lowest one and 0.66 (95% CI: 0.51–0.85) for the highest consumption (*p*-value for trend <0.0001). When considering canned fish intake as a continuous variable, we found an OR of 0.86 (95% CI: 0.79–0.93) for a 10 gr increase in daily intake. These estimates were substantially similar to those derived from models 1, 2, and 3.

When we estimated the risks for colon and rectal cancers separately, an inverse association with canned fish consumption was confirmed in both sites: for colon cancer, the OR for 1 < 2 s/w was 0.82 (95% CI: 0.70–0.95), and for ≥2 s/w, the OR was 0.66 (95% CI: 0.49–0.90) (*p*-value for trend = 0.0004); for rectal cancer, the OR for 1 < 2 s/w was 0.80 (95% CI: 0.67–0.97), and for ≥2 s/w, the OR was 0.65 (95% CI: 0.44–0.95) (*p*-value for trend = 0.0024) (data not shown). Similar results were also found for specific subsites, although larger confidence intervals were found, and the results were no longer statistically significant.

Table 3 shows the ORs (95% CI) of CRC for different types of fish consumption. The consumption of canned fish only with respect to no consumption of fish at all was associated with a decrease of 23% of CRC risk (OR = 0.77, 95% CI: 0.62–0.97). The strongest inverse association was found for the consumption of canned and fresh fish together, with an OR of 0.69 (95% CI: 0.58–0.81).

**Figure 2:**
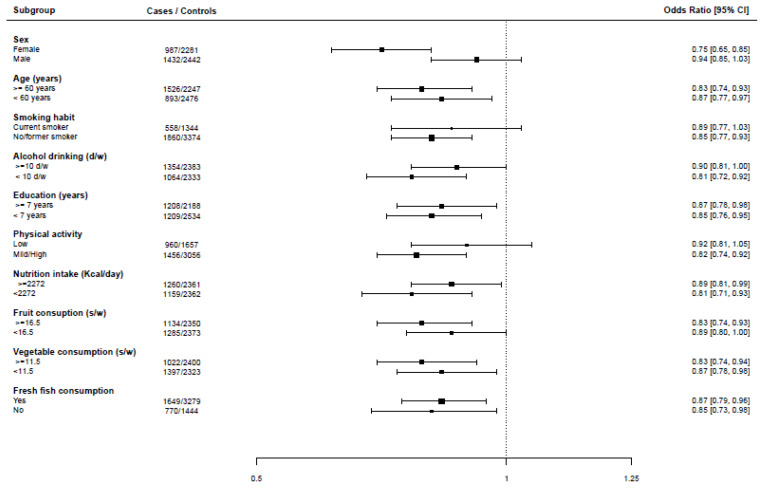
Odds ratios (95% confidence interval) of risk of colorectal cancer according to consumption of canned fish in strata of selected covariates. Legend of Figure 2. d/w: drinks/week; s/w: servings/week.

We also analysed the effect of a 10 gr increase in the daily intake of canned fish across strata of sociodemographic, lifestyle, and dietary factors. The OR was 0.94 (95% CI: 0.85–1.03) in males and 0.75 (95% CI: 0.65–0.85) in females, with a significant interaction between canned fish and sex (*p*-value = 0.011). No other significant interactions between canned fish intake or other factors emerged. The inverse relationship with canned fish was consistently significant in strata of age, smoking habits, alcohol drinking, education, energy intake, vegetable intake, fruit intake, and fresh fish consumption (Figure 2).

## 4. Discussion

We found an inverse association between canned fish consumption and the risk of CRC and colon and rectum cancers separately, which persisted after adjustment for several recognised confounding factors and for the strata of covariates. The risk decreased with an increase in the consumption of canned fish.

Previous studies reported a protective effect of fresh fish on CRC. A case–control study conducted in China found an almost halved risk of CRC for the highest level of fish consumption [18]. In a European study based on the EPIC cohort, and including about half a million participants, the risk of CRC was significantly decreased in the highest quintiles of consumption [19]. However, a prospective cohort study conducted in the United States did not find any significant protective effects of fish and PUFAs on CRC risk in men or women [20]. A meta-analysis conducted by Wu et al. [21], which included a total of 41 studies, found stronger effects in case–control studies than in prospective studies. Thus, inconsistencies were observed across studies considering fish consumption and cancer risk; it is not clear whether such inconsistencies could be explained by unaccounted differences in the fat content of fish, cooking practices across studies, or by the small numbers of cases.

N-3 fatty acids, in particular, the long-chain polyunsaturated fatty acids eicosapentaenoic and docosahexaenoic acids, which are present in cold-water fish and fish oil, may inhibit carcinogenesis [22]. Preclinical studies have indicated that the protective effect of fish on CRC is mainly due to n-3 PUFAs. In vitro studies showed the inhibitory effects of n-3 PUFAs on colon cancer cell lines [23], and in vivo studies showed that n-3 PUFAs suppress chemical-induced colon carcinogenesis in mice and rats [24]. However, the role of fish and n-3 PUFAs in the aetiology of colon and rectal cancer across populations whose fish consumption is high and in which the variation in n-3 PUFA consumption is large remains controversial and yet to be fully elucidated. Other components in fish, however, could also be responsible for its favourable role. Fish consumption is one of the most important sources of selenium, which has been shown to prevent cancer in vivo and in vitro [25].

Generally, canned and fresh fish present similar nutritional characteristics. The industrial process carried out to produce canned fish is likely to preserve the most important nutrients and nutritional properties of fresh fish. For example, the tuna parts selected for canning are rich in fat, and, consequently, they present a high concentration of n-3 PUFAs [26]. Moreover, given that in this study we only considered canned fish in olive oil, we cannot exclude that at least part of the benefits herein observed might be due to olive oil [27].

Our study shows a stronger inverse relationship for women than for men. A cohort study of fresh fish consumption and CRC found similar inverse associations both in men and women [19], and a meta-analysis found an inverse association in men but not in women [5]. In a large Swedish female population-based cohort, the support for an association of n-3 PUFAs with colorectal cancer was small [28].

Canned fish showed a protective role independently of other dietary habits, suggesting that canned fish can be protective *per se*, and its effect was not driven by other selected dietary habits. In particular, canned fish consumption was also inversely related to CRC in individuals who did not consume fresh fish. Interestingly, canned fish had a borderline significant association in subjects with a low intake of fruit and vegetables, suggesting that the lack of an adequate consumption of fruit and vegetables and the consequent effect on CRC risk were not completely blunted by other healthy dietary habits.

In this study, the protective effect of canned fish on CRC substantially equalled that of fresh fish, and the consumption of both types of fish provided an even greater effect, suggesting that fish consumption offers protection from CRC, however processed.

## 5. Limitations and Strengths

The satisfactory reproducibility [15] and validity [14] of the questionnaire is reassuring. Reporting bias, potentially due to some desirability issues, is not likely to have affected the reporting of canned fish consumption in either interviewers or interviewed subjects. Selection bias should not have affected the findings since the cases and controls were comparable in terms of catchment area and hospital. The strengths of this study are the possibility to adjust for several potential confounding factors, the large sample size, and the accuracy of the information collected by a selected group of carefully trained interviewers.

The questionnaire asked about the consumption of tuna, mackerel, and sardines in oil, and, therefore, our results cannot be generalised to canned fish without oil or to other fishes. Indeed, particularly in relation to the time of the first study, canned fish was usually limited to those same fishes in olive oil, and our data should well represent the canned fish consumption of the Italian population in the two periods when the studies took place.

## 6. Conclusions

Overall, few studies considered canned fish, and only a few of them considered fresh fish in addition to canned fish as a unique item [29], therefore preventing disentanglement of their possibly different effects on CRC risk. Since primary prevention remains the key strategy to reduce the increasing global burden of CRC, and given the role of diet in cancer prevention, studying canned fish as a separate food can help identify strategies to support healthy dietary habits in larger strata of the population. This, in turn, could have relevant public health implications, given the high ranking of CRC in incidence and mortality. In Italy, canned fish consumption increased during the 2008 crisis and then during the COVID-19 lockdown, possibly because it was more easily accessible and perceived as more convenient [11,30]. These findings offer a base to support canned fish consumption as a component of a healthy diet.

## Figures and Tables

**Table 1:** Distribution of 2419 colorectal cancer cases and 4273 controls according to selected characteristics.

		Cases (N = 2419)	Controls (N = 4723)	Missing	*p*-Value
Centre					<0.0001
	Pordenone	856 (35.4)	1606 (34.0)		
	Milan	715 (29.5)	1403 (29.7)		
	Genoa	225 (9.3)	498 (10.6)		
	Forlì	94 (3.9)	247 (5.2)		
	Naples	193 (8.0)	387 (8.2)		
	Rome/Latina	336 (13.9)	582 (12.3)		
Sex					<0.0001
	Males	1432 (59.2)	2442 (51.7)		
	Females	987 (40.8)	2281 (48.3)		
Age (years)					<0.0001
	<40	84 (3.5)	359 (7.6)		
	40–50	208 (8.6)	771 (16.3)		
	50–60	601 (24.8)	1346 (28.5)		
	60–70	1018 (42.1)	1579 (33.5)		
	>70	508 (21.0)	668 (14.1)		
BMI (Kg/m^2^)				33	0.9755
	<25	1076 (44.7)	2113 (45.0)		
	25–30	987 (41.0)	1915 (40.7)		
	>30	345 (14.3)	673 (14.3)		
Education (years)				3	<0.0001
	<7	1209 (50.0)	2534 (53.7)		
	7–11	662 (27.4)	1324 (28.0)		
	>12	546 (22.6)	864 (18.3)		
Family history					<0.0001
	Yes	244 (10.1)	192 (4.1)		
	No	2175 (89.9)	4531 (95.9)		
Occupational physical activity at age 30–39				13	0.0009
	Low	960 (39.8)	1657 (35.2)		
	Moderate	806 (33.3)	1698 (36.0)		
	Heavy	650 (26.9)	1358 (28.8)		

**Table 2:** Adjusted odds ratios (ORs) and 95% confidence intervals (CIs) of colorectal cancer according to frequency of canned fish consumption.

Canned Fish Consumption	Model 1	*p*-Value for Trend	Model 2	*p*-Value for Trend	Model 3	*p*-Value for Trend	Model 4	*p*-Value for Trend
<1 serving/week	1	<0.0001	1	<0.0001	1	<0.0001	1	<0.0001
1 < 2 serving/week	0.80 (0.71–0.91)		0.80 (0.71–0.91)		0.80 (0.71–0.91)		0.81 (0.71–0.92)	
≥2 servings/week	0.66 (0.51–0.84)		0.67 (0.52–0.87)		0.66 (0.51–0.85)		0.66 (0.51–0.85)	
10 gr/die	0.87 (0.80–0.94)	0.0003	0.87 (0.80–0.94)	0.0005	0.86 (0.79–0.93)	0.0002	0.86 (0.79–0.93)	0.0002

In model 1, estimates were adjusted for centre, study, sex, and age; model 2 corresponds to model 1 plus BMI, education, and family history of colorectal cancer; model 3 corresponds to model 2 plus physical activity at work, smoking habits, alcohol consumption, vegetable and fruit consumption, and energy intake; model 4 corresponds to model 3 plus fresh fish consumption.

**Table 3:** Adjusted odds ratios * (ORs) and 95% confidence intervals (CIs) of risk of colorectal cancer according to consumption of different types of fish.

Type of Fish Consumption	Cases N (%)	Controls N (%)	OR (95% CI)	*p*-Value
No fish	617 (25.5)	1092 (23.1)	1	
Only canned fish	153 (6.3)	352 (7.5)	0.77 (0.62–0.97)	
Only non-canned fish	1226 (50.7)	2282 (48.3)	0.88 (0.77–1.00)	
Both	423 (17.5)	997 (21.1)	0.69 (0.58–0.81)	<0.0001

* Adjusted for centre, study, sex, age, body mass index, education, family history of colorectal cancer, physical activity at work, smoking habits, alcohol consumption, vegetable and fruit consumption, and energy intake.

## Data Availability

The data described in the manuscript will not be made available in accordance with the indication of the Ethics Committee.
